# cNEUPRO: Novel Biomarkers for Neurodegenerative Diseases

**DOI:** 10.4061/2010/548145

**Published:** 2010-09-19

**Authors:** Philipp Spitzer, Hans Wolfgang Klafki, Kaj Blennow, Luc Buée, Hermann Esselmann, Sanna-Kaisa Herruka, Connie Jimenez, Peter Klivenyi, Piotr Lewczuk, Juan Manuel Maler, Katrin Markus, Helmut E. Meyer, Chris Morris, Thorsten Müller, Markus Otto, Lucilla Parnetti, Hilkka Soininen, Susanna Schraen, Charlotte Teunissen, Laszlo Vecsei, Henrik Zetterberg, Jens Wiltfang

**Affiliations:** ^1^Laboratory for Molecular Neurobiology, Department of Psychiatry and Psychotherapy, University of Duisburg-Essen, LVR-Klinikum Essen, Virchowstraße 174, 45147 Essen, Germany; ^2^Department of Psychiatry and Neurochemistry, Institute of Neuroscience and Physiology, The Sahlgrenska Academy at the University of Gothenburg, 431 80 Mölndal, Sweden; ^3^Inserm U837, 1 Place de Verdun, 59045 Lille, France; ^4^Faculte de Medicine, Université Lille-Nord de France, UDSL, rue Paul Duez, 59800 Lille, France; ^5^CHU, bd. Pr J. Leclerc, 59037 Lille, France; ^6^Department of Neurology, Institute of Clinical Medicine, University of Eastern Finland, Ylipistonranta 1C, 70211 Kuopio, Finland; ^7^OncoProteomics Laboratory, Department of Medical Oncology, VU University Medical Center, De Boelelaan 1117, 1081 HV Amsterdam, The Netherlands; ^8^Department of Neurology, University of Szeged, P.O. Box 427, 6701 Szeged, Hungary; ^9^Department of Psychiatry and Psychotherapy, University of Erlangen, Schwabachanlage 6, 91054 Erlangen, Germany; ^10^Functional Proteomics, Medizinisches Proteom-Center, Ruhr-University Bochum, Universitätsstraße 150, 44780 Bochum, Germany; ^11^Medical Proteomics/Bioanalytics, Medizinisches Proteom-Center, Ruhr-University Bochum, Universitätsstraße 150, 44780 Bochum, Germany; ^12^Medical Toxicology Centre, Institute for Ageing and Health, Institute of Neurosciences, University of Newcastle, Wolfson Unit, Claremont Place, Newcastle upon Tyne NE2 4AA, UK; ^13^Department of Neurology, University of Ulm, Steinhövelstraße 1, 89075 Ulm, Germany; ^14^Clinica Neurologica, Università di Perugia, Ospedale S. Maria della Misericordia, 06156 Perugia, Italy; ^15^Department of Clinical Chemistry, VU University Medical Center, P.O. Box 7057, 1007 MB Amsterdam, The Netherlands

## Abstract

“clinical NEUroPROteomics of neurodegenerative diseases” (cNEUPRO) is a Specific Targeted Research Project (STREP) within the sixth framework program of the European Commission dedicated to the search for novel biomarker candidates for Alzheimer's disease and other neurodegenerative diseases. The ultimate goal of cNEUPRO is to identify one or more valid biomarker(s) in blood and CSF applicable to support the early and differential diagnosis of dementia disorders. The consortium covers all steps required for the discovery of novel biomarker candidates such as acquisition of high quality CSF and blood samples from relevant patient groups and controls, analysis of body fluids by various methods, and finally assay development and assay validation. Here we report the standardized procedures for diagnosis and preanalytical sample-handling within the project, as well as the status of the ongoing research activities and some first results.

## 1. Introduction

The diagnosis of Alzheimer's Disease (AD) is currently based primarily on clinical symptoms. Whereas the sensitivity of the clinical diagnosis for possible and probable Alzheimer Dementia according to National Institute of Neurological and Communicative Disorders and Stroke and the Alzheimer's Disease and Related Disorders Association (NINCDS-ADRDA) criteria is over 80%, its specificity is rather low [1]. The term mild cognitive impairment (MCI) was introduced for subjects who complain about verifiable cognitive disturbances but who show a preserved general cognitive functioning and no impairment in the activities of daily living [2]. These patients can be further subdivided into those with an impaired memory function (amnestic MCI) and those whose memory is preserved but who show disturbances of language, executive function, or visual-spatial skills (Nonamnestic MCI) [2]. If only one of the above-mentioned cognitive domains is impaired, patients are called single-domain MCI; if two or more domains are affected, they are referred to as multidomain MCI. Although the term MCI is solely descriptive and allows no conclusion on the aetiology, the classification allows some prediction of the course of the disease. For amnestic MCI patients, the risk to convert to Alzheimer's Dementia is 10–15% per year [3]. Yet, an accurate early diagnosis in MCI patients or even a predictive diagnosis in individuals without cognitive disturbances is still virtually impossible. As there is evidence that pathological biochemical changes start many years before the occurrence of functional symptoms, identification of biological markers in individuals with early-stage dementia is the most promising way to facilitate a predictive diagnosis [4–6].

Improving the early and predictive diagnosis of AD is of paramount importance if, in the future, preventive and disease-modifying therapies become available. In this regard, enormous efforts are under way. Although most therapies failed to show efficacy in Phase III trials, there are still some promising approaches like A*β* lowering compounds, inhibitors of inflammation, inhibitors of tau phosphorylation and aggregation, and compounds interfering with cholesterol metabolism under investigation [7]. Although the brain has some limited regenerative capacity, neurons are still difficult to replace [8, 9]. Therefore, it is clear that maximal benefit for the patients can be expected when the treatment can be initiated as early as possible in the course of the disease. Furthermore, biologically valid and clinically accurate biomarkers may serve in the development of novel therapeutic strategies and may provide important information in clinical trials of therapies [10].

Well-documented biomarkers for AD in cerebrospinal fluid (CSF) include alterations in A*β*_1-42_, total-tau, and phospho-tau [10]. Importantly, these particular changes are detectable in early dementia stages as well as in individuals with mild cognitive impairment (MCI) who are at high risk of conversion to AD [11]. When analyzed in well-characterized clinical samples, the measurement of A*β*_1-42_, tau, and phospho-tau in cerebrospinal fluid generally allows the diagnosis of AD and even the prediction of the conversion from MCI to AD with a specificity and sensitivity of about 85% [12]. However, some report a lower sensitivity of below 50% for single biomarkers when these biomarkers are measured as part of a routine diagnostic test in a memory clinic [13]. This drop in sensitivity can be explained by the fact that in clinical practice the reference cohort is not a group of cognitively healthy individuals but consists of patients with other neurodegenerative and neurologic diseases who may also have slightly elevated total-tau, phospho-tau, or A*β*_1-42_ levels [13]. The application of these markers in the differential diagnostic of neurodegenerative diseases therefore proves to be particularly problematic [14]. Consequently, there is a need for additional and more sensitive CSF biomarkers for the early and differential diagnosis of Alzheimer's Disease.

There is the additional problem of lumbar puncture to obtain CSF, since although the rate of complications during and after lumbar puncture is below 2–4% and restricted to mild to moderate postlumbar puncture headache [15–18], it must still be seen as invasive method for which special precautions must be taken. Consequently, there is a pressing need for new biomarkers in more easily accessible body-fluids such as peripheral blood.

Clinical proteomics is a fast developing field dedicated to the search for new biomarkers applicable to support the clinical diagnosis [19]. At present, a number of potential new biomarker-candidates for AD have been reported from proteomic studies [20, 21]; unfortunately, however, the published data is often contradictory and in many cases, a solid reassessment by other methods and with independent samples is required [19].

Taking this into account, the EU-project Clinical Proteomics for Neurodegenerative Diseases (cNEUPRO) is not only dedicated to the detection of potential new biomarker candidates for neurodegenerative diseases in CSF and blood, but also to the implementation of in-depth reassessments and validation studies. Finally, promising biomarker candidates will be studied for their suitability as routine test analytes by prototype assays.

## 2. cNEUPRO: The Consortium, Goals, and Workflow

cNEUPRO (http://www.cneupro.eu/) is a Specific Targeted Research Project (STREP) within the sixth framework program of the European Commission. It started in April 2007 and is coordinated by Jens Wiltfang, University of Duisburg-Essen. For the general aims of cNEUPRO, (see Box 1). The consortium consists of 14 academic partners (University of Duisburg-Essen, Centre Hospitalier Universitaire de Montpellier, Sahlgrenska Academy at the University of Gothenburg, VU University Medical Center, University of Ulm, University of Newcastle upon Tyne, University of Aveiro, University of Szeged, University of Perugia, Ruhr-University Bochum, Heinrich Heine University of Duesseldorf, University of Eastern Finland Kuopio, Institut de la Santé et de la Recherche Médicale, University of Erlangen) as well as four small to medium enterprises. (Matrix Advanced Solutions Germany GmbH, MicroDiscovery GmbH, Protagen, BioGenes GmbH).

**Figure 1:**
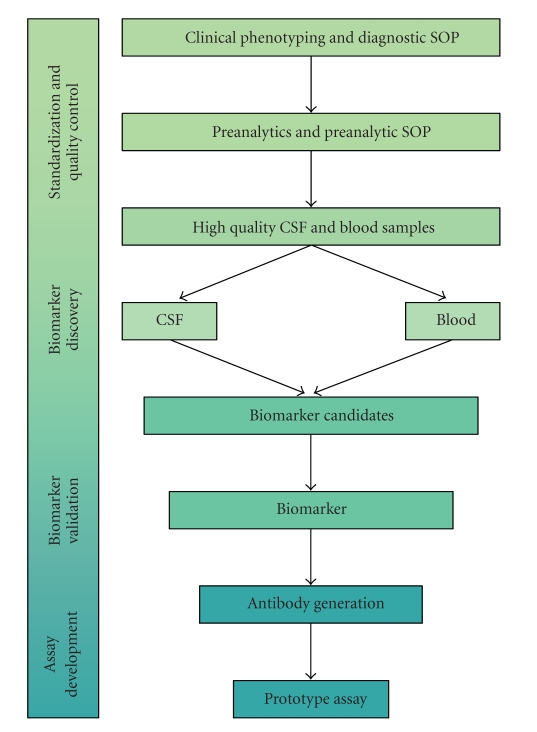
Workflow within the project.

cNEUPRO integrates almost all different levels of biomarker research: the primary phase involves the comprehensive clinical characterization of patients and standardized sample-acquisition and handling by specialized geriatric psychiatrists and neurologists. These samples are subsequently used in the search for candidate biomarkers, their biochemical identification by mass spectrometry, and their reassessment in a second, independent set of high quality samples. Finally, the identified biomarkers will be integrated into novel prototype assays (Figure 1).

The research within cNEURPO concentrates on individuals diagnosed with MCI at baseline who subsequently either developed AD, other dementias, or who did not progress to dementia. As the samples had been taken at baseline, clinical information obtained during follow-up allows the identification of predictive biomarker candidates retrospectively. In addition, clinical samples from patients with early AD at baseline or other dementias in the early stages are also included in the analysis.

In the search for new biomarker candidates in CSF or blood, hypothesis-free proteomic approaches such as urea-based gel electrophoresis, Multidimensional liquid chromatography, combined with two-dimensional differential gel electrophoresis (2D-DIGE), several mass spectrometric methods (e.g., SELDI-TOF, MALDI-TOF, nanoLC-MALDI-TOF/TOF, nanoLC-ESI, nanoLCQFTMS), and array-based methods are conducted. Additionally, specific and potentially interesting molecules are studied in detail in the sense of “hypothesis-driven approaches”. The most promising biomarker candidates will be selected with the aid of biostatistical tools. Where applicable, published information in terms of the biological function or a possible role of selected candidates in the pathophysiology of AD will also be considered. The selected candidates will be reassessed with a further independent high quality clinical sample of age- and sex-matched patients and controls and with assays allowing for intermediate sample throughput and quantitative comparisons. For those biomarker candidates that can be successfully validated, cNEUPRO will devise novel poly- and monoclonal antibodies. Finally the biomarkers will be integrated into novel ELISA-type assays and, if appropriate, in Multiplex-Assays. 

An essential prerequisite for a successful multicenter biomarker-discovery study is the standardization of the clinical diagnostics, the preanalytical sample handling procedures, and the measurements of the known biomarkers total-tau, phospho-tau, and A*β*_1-42_ in CSF. To this end, two neurochemical dementia diagnosis reference centers in Hungary and Portugal are currently being established, and European standard operating procedures for clinical diagnostics and preanalytical sample handling have been defined.

## 3. Current State and First Results of cNEUPRO

### 3.1. Neurochemical Dementia Diagnosis-Reference Center in Hungary Launched

In Hungary, 42 Dementia Centers are responsible for the diagnosis and treatment of demented patients. Before 2009, the CSF analysis of A*β*_1-42_, total-tau, and phospho-tau to support dementia diagnostics was not possible for these centers. As one of the aims of cNEUPRO, the first reference center for neurochemical dementia diagnosis in Hungary was launched in Szeged. With the support of the cNEUPRO consortium, state-of-the-art diagnostic and methodological standards have been implemented, and the center takes part in an ongoing quality control program organized by Kaj Blennow from Sahlgrenska University Hospital, Mölndal, Sweden. During its first twelve months of operation, the neurochemical dementia diagnosis reference center in Szeged has received a total of 54 CSF samples from 14 different Dementia Centers in Hungary. This neurochemical dementia diagnosis center will now try to provide its service to further Dementia Centers in Hungary and to start collecting samples for scientific purposes.

### 3.2. Diagnostic and Preanalytical Standard Operating Procedures

Due to substantial intercenter variations, the reported accuracy of CSF biomarkers is considerably lower in multicenter studies than in single center surveys [22–24]. To this end, a multicenter study, supported by cNEUPRO, provides guidance on how to establish, validate, and audit CSF tau cutoff values using an unbiased, two-stage multicentre strategy [25]. Furthermore, a hands-on workshop was organized by members of the cNEUPRO consortium (paper submitted to the same issue of IJAD). The aim of the workshop was to assess the differences in assay procedures as potential sources of error. During this workshop, 14 groups simultaneously performed the A*β*_1-42_, total-tau, and phospho-tau assays according to the guidelines of the manufacturer. At least 23 items in assay procedures were identified that varied between the laboratories, including procedures for washing, pipetting, incubation, finishing, and sample handling. Thus, even if centers use the same assays for A*β*_1-42_, total-tau, and phospho-tau measurement on a regular basis, they do not uniformly adhere to the procedures recommended by the manufacturer. The results of the workshop stress the importance of standardization of assay protocols. To facilitate biomarker research on a multicenter level, standard operating procedures for the clinical diagnosis and the preanalytical sample handling have been defined by the cNEUPRO consortium (Boxes 2 and 3). The standard operating procedures for sample acquisition, handling, and storage defined by cNEUPRO meet the quality standards required for proteomic studies in CSF [19] and are in agreement with the recently published guidelines for CSF collection and biobanking from the BioMS-eu network [26]. 

### 3.3. Investigated CSF Biomarker Candidates for AD Related to Amyloid Precursor Protein (APP) Processing and Tau Pathology

In the last decade, the levels of A*β* peptides and tau proteins in CSF have gained increasing importance in supporting the clinical diagnosis of AD [10, 33]. As no single marker alone allows for a diagnosis with the desired accuracy, several combinations of CSF-biomarkers (A*β*_*x*-42_, A*β*_*x*-40_, total-tau, phospho-tau) have been proposed [12]. For these markers, a diagnostic accuracy of up to 94% has been achieved in single center studies [12]. Within cNEUPRO, Welge et al. reported a sensitivity and specificity of 88% in the discrimination of AD subjects from other dementias and from elderly depressed individuals with cognitive complaints, by combining the measurement of A*β*_1-40_, A*β*_1-38_, and phospho-tau [34]. With the use of MALDI-TOF mass-spectrometry for the study of CSF samples from AD patients, an oxidized form of A*β*_1-40_ (A*β*_1-40_^ox^) was identified. Quantification by SDS-PAGE/western immunoblot revealed elevated A*β*_1-40_^ox^ levels in patients with AD as compared to probable vascular dementia and controls [35]. Taken together, these pilot studies suggest that besides A*β*_1-42_, additional variants of A*β* peptides may turn out to be specifically altered in AD patients.

Although combinations of these CSF biomarkers were reported to have a high predictive value in single-center studies, their application in multicenter-studies is hampered by relatively high intercenter variations. In an associated multicenter study, including 750 patients with MCI who were followed for at least two years, the conversion to AD could be predicted with a sensitivity of 83% and a specificity of 72% by the ratio of A*β*_1-42_/phospho-tau and total-tau. These values are substantially lower than those seen in several single center studies [24]. The highest intercenter variations were reported for A*β*_1-42_. As this is probably due to its high potential to form aggregates and to stick to test tubes, alternative markers related to APP processing have been investigated within cNEUPRO. In an associated multicenter study, sAPP*α* and sAPP*β*, two proteins secreted in the CSF after the *α*- or *β*-secretase cleavage of APP, were assessed in 188 patients with MCI or mild to moderate AD. In previous studies, sAPP*α* and sAPP*β* were found to be unchanged [36, 37] or decreased [38–40] in the CSF of AD patients. Within cNEUPRO, sAPP*α* and sAPP*β* levels in CSF of MCI and AD patients with elevated total-tau and reduced A*β*_1-42_ CSF concentrations were compared to those from patients without a respective CSF biomarker profile. Both were found to be higher in the CSF from patients with an AD-indicative biomarker profile [41]. Taken together, these results suggest that sAPP*α* and sAPP*β* may be indicators of altered APP expression and/or metabolism. Reports on their value as candidate biomarkers are however so far contradictory.

In a different study which was supported by cNEUPRO, six novel N-terminal APP-fragments with molecular masses of approximately 12 kDa and starting at amino acid 18 of the APP sequence were detected in CSF by mass spectrometry. In a subsequent small pilot study, six of six AD patients and five of five controls could be classified correctly by the combined evaluation of five of the six fragments [42]. Additionally, Immuno-MS analysis of CSF has led to the detection of eleven novel APP fragments, which begin N-terminally to the *β*-secretase cleavage site, and end one amino acid before the proposed *α*-secretase cleavage site (APP/A*β* peptides) [43]. Interestingly, seven of the twelve APP/A*β* peptides were significantly upregulated in AD [43].

### 3.4. CSF-Biomarker Candidates for AD Investigated within cNEUPRO, Which Are Not Related to APP Processing or Tau Pathology

One of several kinases that have been suggested to be involved in the abnormal hyperphosphorylation of tau is the MAP-kinase ERK1/2. In a methodological pilot study, ERK 1/2 and its doubly phosphorylated, activated form have been detected in a small number of CSF samples from patients with AD, MCI, and frontotemporal lobar degeneration (FTLD) [44]. To evaluate the usefulness of ERK 1/2 as a potential novel CSF biomarker, ERK1/2 levels in CSF are currently being studied in a total of 110 CSF samples from partners within the consortium with a chemiluminescent 96 well assay format.

In accordance with a previous report [45], research within cNEUPRO found glial fibrillary acidic protein (GFAP), a marker for astrogliosis, to be increased in CSF of AD and sporadic Creutzfeldt-Jacob Disease (sCJD) patients. CSF samples of 18 AD patients, 22 sCJD cases, and 18 from nondemented controls were analyzed with the use of a commercially available ELISA. In AD, a remarkable elevation in CSF GFAP levels with no overlap to controls was observed. Although a significant increase in GFAP could be observed in CJD as well, this was not as pronounced as in AD [46]. Consequently GFAP might have some additive value as part of a biomarker supported diagnosis, although it lacks specificity for AD.

Chronic inflammation associated with oxidative and nitrosative stress is another aspect which is considered to be important in the pathophysiology of AD [47]. The most common protein markers of oxidative and nitrosative stress are protein-bound carbonyls and 3-nitrotyrosine [48]. An increased oxidation of certain proteins and an increased concentration of 3-nitrotyrosine have been reported in tissue [49] and CSF [50–52] of AD patients, but there is also contradictory data indicating no difference between AD and controls [53]. In a study conducted by members of the cNEUPRO consortium, where the concentrations of 3-nitrotyrosine and total protein carbonylation were measured, no change was found in CSF of AD patients [48]. Yet, slightly reduced levels of protein carbonyls were detected in ApoE-*ε*4 carriers as compared to ApoE-*ε*4 noncarriers [48]. These results suggest that the concentrations of total protein carbonyls and 3-nitrotyrosine are at this stage not suitable to monitor the chronic inflammatory processes related to AD.

### 3.5. Investigated CSF Biomarkers for Other Neurodegenerative Diseases

In addition to promoting the early and predictive diagnosis of AD, cNEUPRO is also dedicated to search for new biomarkers to support the diagnosis of other neurodegenerative diseases such as sCJD, FTLD, vascular dementia (VaD), Dementia with Lewy bodies (DLB), Parkinson's Disease (PD), and Parkinson's Disease Dementia (PDD).

Two-dimensional differential gel electrophoresis (2D-DIGE) followed by MALDI-TOF mass-spectrometry indicated that CSF from patients with sCJD differed from CSF from patients with other neurological deficits on the basis of several protein spots. Among these, several previously identified surrogate markers of sCJD such as 14-3-3 protein, neuron-specific enolase, and lactate dehydrogenase were identified. Additionally, an unidentified protein of 85 kDa was found to be significantly increased in sCJD patients [54].

In a separate cNEUPRO investigation, SELDI-TOF mass spectrometry was applied in the analysis of CSF from 32 sCJD patients, 32 controls, and 31 patients with other dementias. Ubiquitin, an 8.6 kDa protein involved in protein degradation, was found to be elevated in the CSF of sCJD cases. This could be confirmed by reassessment with western immunoblots. In the study population, the accuracy of a biomarker-based classification of the samples could be significantly improved by including Ubiquitin in addition to tau, and 14-3-3 protein [55]. This finding is in accordance with several previous reports where Ubiquitin was also found to be elevated in the CSF of sCJD patients [56]. As there is also evidence for altered levels of CSF Ubiquitin in AD [57–59] and vascular dementia [60], it seems that this observation is related to neurodegenerative processes in general and not to a specific disease. Yet, in the Steinacker study CSF Ubiquitin levels in sCJD were higher than those in other dementias [55]. Therefore, Ubiquitin may still be a good biomarker for sCJD if, as with tau protein [61], disease-specific cut-off values are applied.

S100B, another astroglial marker, may also be useful to support the diagnosis of sCJD. Within cNEUPRO, S100B was measured in 54 CSF samples from patients with sCJD, AD, and control patients with the use of a commercial ELISA. Supporting previous findings [62, 63], S100B was shown to be highly elevated in sCJD with no overlap to the other groups [46]. Others have found elevated S100B in familial CJD cases [64], but also in CSF [65] and serum [66] of AD patients. These findings suggest that more attention might be paid to the use of astroglial markers in supporting the differential diagnosis of dementias [46].

With respect to FTLD, cNEUPRO found elevated mean levels of the TAR DNA-binding Protein 43 (TDP-43) and reduced A*β*_1-42_ levels [67, 68]. In line with the reported increased gene expression of TDP-43 in brain tissues [69], elevated 45 kDa TDP-43 levels were found in the CSF of 12 patients with FTLD as compared to 13 nondemented controls by western-immunoblot [67].

In the same sample, the assessment of different A*β* peptide species, sAPP*α* and sAPP*β*, by electrochemiluminescence-based multiplex assays indicated no significant difference for sAPP*α* and sAPP*β* between the groups. However, reduced A*β*_1-42_ levels were found in FTLD [68]. These findings are supported by several earlier studies which found CSF-levels of A*β*_1-42_ in FTLD to be lower than in nondemented controls and higher than in AD [70–73]. However, there are also contradictory publications, regarding levels of A*β* species which did not find reduced CSF A*β*_1-42_ concentrations in FTLD [74, 75]. Although TDP-43 and fragments of APP processing are currently not suitable as biomarkers because of a large overlap between the different diagnostic groups, these findings may still reflect aspects relevant for understanding the pathophysiology of these disorders.

In an associated study focussed on the biomarker supported differential diagnosis of AD, PD, PDD, and DLB, CSF A*β*_1-42_, total-tau, and phospho-tau were measured in the CSF of a total of 80 patients. Although some significant differences in the average biomarker measurements were found between the groups, only AD patients could be effectively differentiated from patients with other dementias by phospho-tau. For A*β*_1-42_, total-tau, and phospho-tau, a large overlap between the other neurodegenerative diseases was observed. Interestingly, only in DLB were A*β*_1-42_ and total-tau found to correlate with the duration and the severity of dementia [76]. Consequently, more and better biological markers are needed to support the differential diagnosis of these dementias [77].

A marker with a potential specificity for synucleinopathies may be the lysosomal hydrolase *β*-glucocerebrosidase. In addition to a previous report linking a reduced activity of *β*-glucocerebrosidase to PD [78], a reduced activity of *β*-glucocerebrosidase was specifically found in DLB within cNEUPRO. In CSF from nondemented controls, patients with AD or FTLD, no differences in *β*-glucocerebrosidase activity were found. In contrast, the activity of *α*-mannosidase, another lysosomal hydrolase, was found to be significantly reduced in all investigated neurodegenerative diseases as compared to controls [79]. In order to support the hypothesis that CSF *β*-glucocerebrosidase activity might be a novel CSF biomarker of synucleinopathies, the data need to be confirmed in larger studies.

### 3.6. Investigated Blood-Biomarker Candidates Related to APP Processing

Several recent studies aimed at identifying AD biomarkers in blood were specifically targeted at determination of A*β* peptides in blood plasma or serum [20].

Within a cNEUPRO associated substudy of the German Kompetenznetz Demenzen (http://www.kompetenznetz-demenzen.de/), A*β*_1-40_ and A*β*_1-42_ were assessed in blood plasma from 257 individuals with multiplexing technology on the Luminex platform. A statistically significant decrease of the A*β*_1-42/1-40_ ratio was found in the plasma of the patients with early AD and MCI of AD type whose clinical diagnoses were backed up by corresponding findings in the CSF [80]. Moreover, the cNEUPRO associated French “Three-City study” found that a reduction of the ratios A*β*_1-42_/A*β*_1-40_ as well as A*β*_*x*-42_/A*β*_*x*-40_ was associated with an increased risk of developing dementia within the next two years [81]. In contrast, several other published studies have not reported significant differences in A*β* peptide concentrations in blood plasma between AD patients and controls [82–84]. In summary, there is no definitive conclusion as to whether plasma A*β* reflects the changing level of central amyloid [20]. Due to the substantial interindividual variations and a large overlap between the diagnostic groups, measuring the individual concentrations of A*β* peptides in plasma is not suitable to support the clinical diagnosis of different dementia disorders. However, there is preliminary evidence that specific forms of A*β* peptides in plasma prove to be helpful in the differential diagnosis of AD and other dementias. In a retrospective pilot study which was supported by cNEUPRO, vascular dementia could be differentiated with a sensitivity and specificity of >80% from other dementias and depressive controls by the ratio of A*β*_1-38_/A*β*_1-40_ [85].

Currently, highly sensitive assays for the detection of A*β* peptides in blood and CSF are available for A*β*_*x*-38_, A*β*_*x*-40_, and A*β*_*x*-42_. For a detailed analysis of additional variants of A*β* peptides in blood plasma, a highly sensitive two-dimensional gel separation method was established within cNEUPRO. Using this method, at least 30 different A*β* peptides were observed [86]. Semiquantitative analysis revealed that the peptides A*β*_1-40_ and A*β*_1-42_ accounted for less than 60% of all A*β* peptides that were detected by the specific antibody that was used in this study. At least 10% of the detected A*β* peptides appear to be N-terminally truncated [86]. One possible source of these N-terminally truncated A*β* peptides detected in human plasma is mononuclear phagocytes. Cultures of human mononuclear phagocytes were shown to secrete complex A*β* peptide patterns characterized by a high proportion of N-terminally truncated variants [87]. Furthermore, the secretion of A*β* peptides from human mononuclear phagocytes was differentially regulated in response to cell culture conditions [87] and was elevated in cell cultures of mononuclear phagocytes from AD patients as compared to controls [88]. Additional work is under way to evaluate several N- and C-terminally truncated A*β* peptides in plasma as potential biomarkers for AD.

### 3.7. Currently Ongoing Research in cNEUPRO

The identification of valid biomarkers in blood is highly desirable because they have the advantage of being easily accessible. The search for potential biomarker candidates in plasma or serum is complicated by the presence of a number of highly abundant proteins. These proteins which are believed to have only small diagnostic potential make up about 90% of the whole plasma proteome [89]. As a first step towards biomarker discovery in serum, it was shown that the depletion of 12 high abundant serum proteins by immuno affinity chromatography columns resulted in an increased number of detected peaks by subsequent analysis with SELDI-TOF mass spectrometry [90]. In contrast, CSF proteomics for biomarker discovery in neurodegenerative diseases is particularly attractive because of the proximity of CSF to the brain. Again, the removal of highly abundant proteins resulted in an improved detection of low abundant CSF proteins including brain-derived proteins. Additional separation procedures were introduced to account for the large dynamic range of the expression levels and to simplify the analysis of proteolytically generated peptides by mass spectrometry. For a comparative analysis of individual clinical samples and for a relatively in-depth search for potential novel biomarkers, reproducibility is an absolute requirement. Therefore, different multiaffinity depletion methods followed by gel-nanoLC-MS/MS and spectral counting have been evaluated for the in-depth, label-free quantitative analysis of CSF. Depletion in spin-filter format, coupled to gel-LC-MS/MS, provided a robust method that yielded ~800 CSF proteins per analyzed sample, with acceptable reproducibility of protein identification (71%–74% in technical replicates) and quantification (17%–18% CV on spectral counts). To control for reproducibility, the same workflow was implemented in two separate laboratories within cNEUPRO. This proteomics approach was subsequently applied in both laboratories to the independent analysis of two separate cohorts of 20 individual CSF samples each. In both cohorts the patients were clinically diagnosed, and CSF was taken according to the cNEUPRO standard operating procedures. Both discovery sets of samples included CSF samples from five control subjects, from five subjects with mild cognitive impairment without conversion to AD, from five patients with mild cognitive impairment with conversion to AD within the follow-up of 2 years, and five patients with AD. Both datasets contained ~1100 identified proteins with a total of ~1600 unique CSF proteins in the common dataset and an overlap of ~500 between the two laboratories. The biostatistical analysis is currently on-going to select the most promising candidates for a reassessment by targeted mass spectrometry and antibody-based methods in a larger set of samples.

## 4. Conclusion

Within the first two years, cNEUPRO confirmed sAPP, various A*β* peptide variants, GFAP, S100B, and ubiquitin as biomarker candidates known from previous studies. Additionally, further APP fragments were discovered and TDP-43 as well as *β*-glucocerebrosidase and ERK 1/2 were proposed as potential novel candidate biomarkers for the early and differential diagnosis of neurodegenerative diseases (Table 1). Because of the high complexity of the blood proteome and probably because of its distance from brain pathology, novel biomarkers in serum or plasma are still elusive. To promote biomarkers in support of the clinical diagnosis of neuropsychiatric disorders in Europe, cNEUPRO devised European standard operating procedures for preanalytical sample handling and established a neurochemical dementia diagnosis reference center in Hungary. cNEUPRO has now started to select the most promising biomarker candidates from two proteomic studies within cNEUPRO and to reassess the most promising biomarker candidates with larger sample size and independent methods to finally integrate them into novel prototype assays.

To increase the accuracy of a biomarker-based diagnosis, biomarkers in body-fluids have been combined with other biological markers such as structural and functional neuroimaging and neuropsychological testing [91]. Whether the new biomarker assays which will be developed within cNEUPRO will be useful in such a multimodal diagnostic workup remains to be elucidated.

## Figures and Tables

**Box 1:**
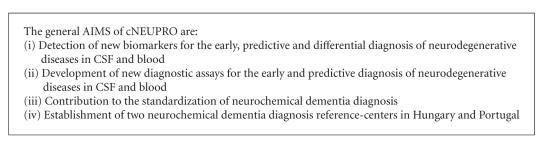
General aims of cNEUPRO.

**Box 2:**
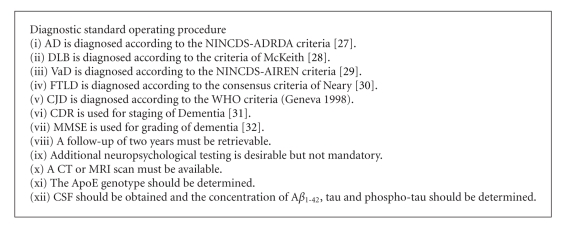
Diagnostic standard operating procedure. AD = Alzheimer's Disease, DLB = Dementia with Lewy-bodies, VaD = Vascular Dementia, FTLD = frontotemporal lobar degeneration, CJD = Creutzfeldt-Jacob Disease, CDR = Clinical Dementia Rating Scale, MMSE = Mini Mental Status Examination. References in the box: [27–32].

**Box 3:**
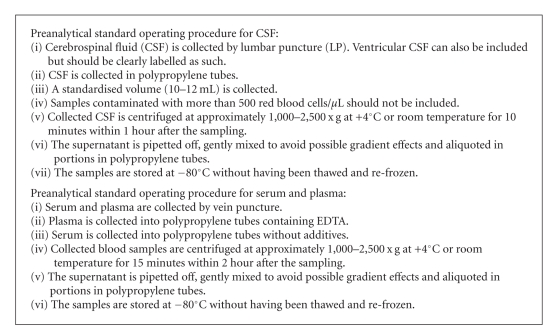
Preanalytic standard operating procedures for CSF and blood.

**Table 1:** List of candidate biomarkers investigated in the context of cNEUPRO. CON: control patient, AD: Alzheimer's Disease, OD: other dementia, VaD: vascular dementia, MCI: Mild cognitive impairment, sCJD: sporadic Creutzfeldt-Jacob Disease, FTLD: Frontotemporal lobar degeneration, ALS: Amyotrophic Lateral Sclerosis, DLB: Dementia with Lewy bodies.

Biomarker candidate	Context/Function	Method	Patients	*n*	Result	Ref.
Investigated CSF candidate biomarkers for AD related to APP processing						

A*β*_1-42/1-38_ ratio	APP processing	ELISA/MSD	CON	30		
			AD	44	Reduced in AD	[34]
			OD	87		

A*β*_1-40_^ox^	APP processing	Western blot	CON	30		
			AD	30	Elevated in AD	[35]
			VaD	37		

sAPP	APP processing	Luminex	MCI	81	Elevated sAPP*α*/*β* in	
			AD	69	patients with elevated	[41]
			OD	38	tau and reduced A*β*_1-42_	

APP/A*β*	APP processing	LC-MS	CON	3	Elevated in AD	[43]
			AD	3		

12 kDa sAPP	APP processing	LC-FTICR-MS	CON	6	Elevated in AD	[42]
			AD	5		
		Western blot	CON	6	Elevated in AD	[42]
			AD	6		

Investigated CSF candidate biomarkers for AD not related to APP processing						

GFAP	Marker for astrogliosis	ELISA	CON	12		
			AD	18	Elevated in AD	[46]
			sCJD	22		

Total protein	Neuro-inflammation	ELISA	CON	18	No difference between	[48]
carbonylation			AD	22	AD and CON	

3-nitrotyrosine	Neuro-inflammation	ELISA	CON	18	No difference	[48]
			AD	22	between AD and CON	

ERK 1/2	MAP-Kinase		MCI	9		
		western blot/electrochemi-luminescence	AD	4	Pilot study, no statistics	[44]
			FTLD	2		

Investigated CSF candidate biomarkers for other dementias						

S100B	Marker for astrogliosis	ELISA	CON	12		
			AD	18	Elevated in sCJD	[46]
			sCJD	22		

TDP-43	DNA binding protein	Western blot	CON	13		
			FTLD	12	Elevated in FTLD	[67]
			ALS	15	and ALS	
			ALS+FTLD	9		

85 kDa protein	Unknown	2D-DIGE/MALDI-TOF	CON	6		
			AD	24	Elevated in sCJD	[54]
			sCJD	36		
			DLB	6		

Ubiquitin	Protein degradation	LC-MS/WB	CON	32		
			sCJD	32	Elevated in sCJD	[55]
			OD	31		

*α*-Mannosidase	Lysosomal Hydrolase	Enzyme activity assay	CON	23		
			AD	20	Reduced in all	[79]
			FTLD	20	dementias	
			DLB	17		

*β*-Glucocerebrosidase	Lysosomal Hydrolase	Enzyme activity assay	CON	23		
			AD	20	Reduced in DLB	[79]
			FTLD	20		
			DLB	17		
